# The significance of dynamin 2 expression for prostate cancer progression, prognostication, and therapeutic targeting

**DOI:** 10.1002/cam4.168

**Published:** 2013-12-18

**Authors:** Bin Xu, Liang Hong Teng, Sabrina Daniela da Silva, Krikor Bijian, Samir Al Bashir, Su Jie, Michael Dolph, Moulay A Alaoui-Jamali, Tarek A Bismar

**Affiliations:** 1 Segal Cancer Center and Lady Davis Institute for Medical Research, Department of Oncology and Medicine, McGill UniversityMontreal, Quebec, Canada; 2 Department of Pathology and Laboratory Medicine, University of Calgary and Calgary Laboratory ServicesEdmonton, Alberta, Canada; 3 Department of Applied Biosciences, Jordan University of Science & TechnologyIrbid, Jordan; 4 Southern Alberta Cancer Institute and Tom Baker Cancer CenterEdmonton, Alberta, Canada

**Keywords:** Cancer-specific mortality, castration resistance, dynamin, prostate cancer, therapeutic target

## Abstract

Dynamin 2 (Dyn2) is essential for intracellular vesicle formation and trafficking, cytokinesis, and receptor endocytosis. In this study, we investigated the implication of Dyn2 as a prognostic marker and therapeutic target for progressive prostate cancer (PCA). We evaluated Dyn2 protein expression by immunohistochemistry in two cohorts: men with localized PCA treated by retropubic radical prostatectomy (*n* = 226), and men with advanced/castrate-resistant PCA (CRPC) treated by transurethral resection of prostate (TURP) (*n* = 253). The role of Dyn2 in cell invasiveness was assessed by in vitro and in vivo experiments using androgen-responsive and refractory PCA preclinical models. Dyn2 expression was significantly increased across advanced stages of PCA compared to benign prostate tissue (*P *<* *0.0001). In the CRPC cohort, high Dyn2 was associated with higher Gleason score (*P *=* *0.004) and marginally with cancer-specific mortality (*P *=* *0.052). In preclinical models, Dyn2 gene silencing significantly reduced cell migration and invasion in vitro, as well as tumor size and lymph node metastases in vivo. In isolated PCA cells, Dyn2 was found to regulate focal adhesion turnover, which is critical for cell migration; this mechanism requires full Dyn2 compared to mutants deficient in GTPase activity. In conclusion, Dyn2 overexpression is associated with neoplastic prostate epithelium and is associated with poor prognosis. Inhibition of Dyn2 prevents cell invasiveness in androgen-responsive and -refractory PCA models, supporting the potential benefit of Dyn2 to serve as a therapeutic target for advanced PCA.

## Introduction

Prostate cancer (PCA) is the second most common cause of cancer-related deaths in men. Although mortality has fallen by 25% over the last decade, and the 5-year survival rate for localized disease is now approaching 100% 1, metastatic and castrate-resistant PCA (CRPC) remain a major cause of cancer-specific deaths. Currently, it is difficult to reliably differentiate between aggressive and indolent forms of PCA prior to treatment implementation. Increasing efforts have been made to identify genomic and proteomic alterations associated with PCA progression, and in few of those studies, dynamin (Dyn) 2, a member of the large GTPase superfamily was identified among genes found to be differentially expressed between benign and PCA tissues 2,3. However, the significance of Dyn2 overexpression in PCA has not been fully addressed.

Dyn is a 96-kDa GTPase originally identified as a microtubule-binding protein and subsequently found to contain a GTP-binding domain 4,5. It comprises three isoforms: Dyn1, which is localized primarily to the central nervous system, and predominantly concentrated in the pre-synapse; Dyn2, which exhibits ubiquitous tissue distribution; and Dyn3, which is predominantly expressed in the testis and the nervous system postsynapse 6. Dyn2 is implicated in a broad range of physiological functions, particularly in the regulation of membrane trafficking where it represents an essential component of vesicle formation in receptor-mediated endocytosis, synaptic vesicle recycling, caveolae internalization, and vesicle trafficking in and out of Golgi apparatus 7,8. Dyn2 plays a dual role in clathrin-mediated endocytosis, functioning at early stages to regulate clathrin-coated pit maturation and at later stages to directly catalyze membrane fission and clathrin-coated vesicle formation 9. In addition to its role in the scission of coated vesicles, Dyn2 regulates invagination of clathrin-coated pits 10 and interacts with additional proteins, including microtubules, to regulate cytokinesis 11.

Mutations in the Dyn2 gene have been linked to inherent rare diseases such as centronuclear myopathy, Charcot–Marie–Tooth type B, as well as Alzheimer's disease and cancer 12–15. In cancer, both enzymatic activity and proper localization of Dyn2 are required for extracellular matrix degradation by invasive cancer cells 15. Dyn2 is also necessary for the endocytosis of several proteins associated with cancer motility and invasiveness, including integrin *β*-1 and focal adhesion (FA) kinase 16,17.

In this study, we investigate Dyn2 expression across various stages of PCA progression, including benign prostate tissue (BN) high-grade prostatic intraepithelial neoplasia (HGPIN), localized PCA and aggressive PCA (CRPC). The prognostic significance of Dyn2 in PCA patients is also assessed. Moreover, the association between Dyn2 expression and PCA invasion and metastasis was characterized using in vitro and in vivo experiments in androgen-responsive and androgen-resistant PCA models.

## Materials and Methods

### Study population

The study population consisted of two cohorts; the first consisted of 226 men with localized PCA treated by retropubic radical prostatectomy as initial monotherapy. Clinical progression was defined as a postoperative serum PSA level increase of >0.2 ng/mL assessed on two separate occasions. Data related to biochemical recurrence were available for 188 patients. The clinical characteristics of this cohort are summarized in Table 1. The second cohort consisted of 253 men with CRPC. Tissue samples for this cohort were obtained by transurethral resection of prostate (TURP). Patients in this cohort were diagnosed with CRPC or advanced disease and they were either managed expectantly or treated by radiotherapy or hormonal therapy. Gleason grading was scored according to the International Society of Urological Pathology 18 and tumor staging done using the TNM classification of malignant tumors. Pathological TNM system in CRPC samples defines pT1a as tumor volumes ≤5% and pT1b as >5%. Follow-up data were obtained through the Alberta provincial cancer registry and reported for all patients. Deaths were divided into those related to PCA or other causes. Disease-specific survival was calculated from the date of TURP procedure to mortality date or last follow-up. Patients were considered to have PCA-specific mortality if they had documented advanced clinical pathological or radiological evidence of disease while on anti-hormonal therapy. Deaths, lacking these criteria, were not considered to have been attributable to PCA. Patients alive at the time of data collection were censored. In this cohort, 241 patients with confirmed clinical outcome data were available for analysis with mean follow-up of 30 months (range 0–60 months). Table 2 shows patients demographics of this cohort relative to Dyn2 expression. All clinicopathological data were obtained with approval of institutional review boards.

**Table 1:** Clinicopathological characteristics of the localized PCA patients.

Variables	Category	
Age (years)	*N*	226
	Mean (SD)	64.0 (6.1)
	Range	(42.7, 80.5)
Gleason score	<7	69 (30.5)
	=7	130 (57.6)
	>7	27 (11.9)
Pathological stage*	pT2	142 (65.4)
	pT3	74 (34.1)
	pT4	1 (0.5)
Surgical margin*	Negative	120 (53.1)
	Positive	97 (42.9)
PSA failure	Yes	62 (33.0)
	No	126 (67.0)
Follow-up time (in years)*	*N*	188
	Mean (SD)	4.8 (3.5)
	Range	(0.0, 15.8)

* Not all patients’ information is available.

**Table 2:** Association of Dyn2 expression in the CRPC cohort.

		Dyn2 expression		
Variables	Category	*P-*value	Negative/weak	Moderate/high
Cases (*N*)		108	145	
Age	(years) (mean; range)	78.81 (55–96)	79.25 (60–97)	0.702
Gleason score				0.004
	<7	32 (30%)	36 (25%)	
	7	29 (27%)	20 (14%)	
	3 + 4	20 (19%)	11 (8%)	
	4 + 3	9 (8%)	9 (6%)	
	>7	47 (43%)	89 (61%)	
# of PCA deaths*		15/96 (15.6%)	38/145 (26.2%)	0.052

* Not all patients’ information is available. PCA, prostate cancer.

### Tissue microarray construction

Tissue microarray (TMA) blocks were created using a manual tissue arrayer (Beecher Instruments, Silver Spring, MD) as previously described 19. For each case, cores from multiple areas were used. HGPIN and adjacent benign tissue were also sampled (when applicable). After construction, 4 *μ*m sections were initially cut and stained with hematoxylin and eosin to verify the histological diagnosis.

### Dyn2 immunohistochemistry

The slides were incubated for 60 min at 37°C using Ventana autostainer with Dyn2 polyclonal antibody (H-300; Santa Cruz Biotechnology Inc., Santa Cruz, CA; 1:25). A Ventana iView DAB detection kit (Ventana, Tucson, AZ) was used for HRP detection and counterstain. A multi-tissue control TMA slide was used as control. Negative controls were performed by substituting the primary antibody with pre-diluted normal mouse serum at a dilution of 1:200.

### Pathologic analysis

TMA cores were assigned a diagnosis by two certified pathologists. Protein expression intensity was evaluated semiquantitatively without prior knowledge of any clinical information using a four-tiered system (0, negative; 1, weak; 2, moderate; and 3, strong) as previously described 19. Each core was evaluated separately and a final score for each case was achieved by averaging the total intensity value of all cores within a specific patient sample.

### Statistical analysis

Subject characteristics were presented as means, standard deviations, and ranges for continuous variables, and frequencies and percentages for categorical variables. Tukey test was used to compare the difference in Dyn2 expression and pathological diagnoses, followed by multiple comparison tests. Chi-square test or Fisher's exact test was used to test for associations between the status of Dyn2 and other key variables such as diagnosis, pathological stage, surgical margin, and tumor grade. For the time to cancer-specific mortality, the Kaplan–Meier approach along with the log-rank test was used to explore the association between Dyn2 expression and cancer-specific deaths. All reported *P*-values are two-tailed and were considered significant if <0.05.

### Cell lines and cell culture

The PCA cell lines LNCaP and PC3 were originally obtained from ATCC (Manassas, VA). The castration-resistant C4-2 PCA cell line derived from the parental LNCaP cell line was kindly provided by Dr. N. Zoubeidi (The Prostate Centre, Vancouver General Hospital, University of British Columbia) 20. The highly invasive PC3M cell variant was provided by Dr. I. Fidler (M. D. Anderson Cancer Center) 21. These cells were maintained in culture in RPMI medium (Life Technologies, Rockville, MD) supplemented with 10% fetal bovine serum (FBS) and 1% penicillin/streptomycin. The non-immortalized normal prostate epithelial cells (PrEC) were from Clonetics (Bulletkit; Clonetics Corporation, San Diego, CA) and maintained in their proprietary serum-free media.

### Dyn2 silencing and reconstitution

A 19-nucleotide sequence used to target human Dyn2 mRNA: GACATGATCCTGCAGTTCA 22 and scrambled negative control sequence were cloned into pSuper-retro puromycin vector according to the manufacturer's instructions (Oligoengine, Seattle, WA). After packaging in Phoenix cells, the retrovirus contains either control scrambled shRNA (PSR [pSUPER retro vector]) or Dyn2 shRNA were collected to infect target cells as we described previously 23. For reconstitution experiments, Dyn2 silenced PC3 cells were transfected with either wild-type Dyn2 (siRNA + WT), a GTPase-deficient mutant (siRNA + K44A), a proline-rich domain (PRD) mutant in which the entire C-terminal PRD was deleted (siRNA + ΔPRD) or a pleckstrin-homology (PH) domain mutant where a substitution of lysine 535 to alanine was introduced (Dyn2 + K535A) 24.

### Western blot analysis

Cell lysates were boiled and separated using 10% sodium dodecyl sulfate polyacrylamide gel electrophoresis (SDS PAGE) gel as we described earlier 25. Membranes were immunoblotted with anti-Dyn2 (H-300; Santa Cruz Biotechnology Inc.; 1:200) and anti-glyceraldehyde 3-phosphate dehydrogenase (GAPDH) (clone 6C5; Cedarlane Laboratories, Ontario, Canada; 1:2000).

### Cell proliferation and invasion assay

Exponentially growing cells (1 × 10^3^) were seeded in 96-well plates in complete medium and cell survival was evaluated 72 h later using the 3-[4,5-dimethylthiazol-2-yl]-2,5 diphenyl tetrazolium bromide metabolic assay 26. Cell invasion assay was performed using 8 *μ*m porous chambers coated with Matrigel (Becton Dickinson, Bedford, MA) according to the manufacturer's recommendations 23. Each experiment was performed at least in quadruplicate and results expressed as mean ± SD. Statistical significance was analyzed using Student's *t*-test.

### Immunofluorescence microscopy

Immunofluorescence assay was performed as previously described 27. Briefly, cells were seeded on coverslips then fixed with paraformaldehyde, followed by blocking and incubated with the following primary antibodies: Dyn2 polyclonal antibody (H-300; Santa Cruz; 1:100) and monoclonal anti-vinculin (clone hVIN-1; Sigma-Aldrich, Steinheim, Germany; 1:200). Cells were then incubated with secondary antibodies conjugated to Texas Red or Cy2 (Jackson Immunoresearch Laboratories, West Grove, PA). After mounting with gelvatol medium (Airvol®205 polyvinyl alcohol, Air products and Chemicals, Inc., Allentown, PA), coverslips were analyzed using a fluorescent microscope (Axiophot; Carl Zeiss MicroImaging, Inc.).

### Analysis of FA dynamics in live cells

FA turnover was investigated using a real-time imaging by confocal microscopy as described earlier 27. Briefly, cells were transfected with EYFP-paxillin (a FA marker) and plated into a multi-well chambered cover glass (LabTek, Nalge Nunc International, Rochester, NY). After starvation, the cells were stimulated with 10 ng/mL epidermal growth factor. Fluorescent images were captured every 4 min for 2 h using Nipkow spinning disk confocal microscope (WaveFx spinning disk; Quorum Technologies Inc., Guelph, Canada). For quantification of FA protein dynamics, fluorescence intensities of individual adhesions from background-subtracted images were measured over time using Volocity imaging software (PerkinElmer, Waltham, MA) and quantified as we described earlier 27. For each condition, measurements were made on at least 25 individual adhesions in 10 separate cells. Duration measurements were made for same adhesions by counting the amount of time lapsed between the first and last frames in which an individual adhesion was observed. Data on graphs are presented as mean ± SD. Statistical significance was evaluated using Student's *t*-test.

### In vitro and in vivo studies in PCA preclinical models

In vivo studies were approved by Institutional Animal Care Committee. Male SCID mice were obtained from Charles River Laboratories (St. Zotique, PQ, Canada). PC3 cells (1 × 10^6^ cells) expressing Dyn2 shRNA (siRNA-Dyn2) or scrambled shRNA (PSR) were injected orthotopically into mice prostates (*n* = 10). The cell lines LNCaP and C4-2 were washed twice in PBS and then mixed with matrigel (Becton Dickinson) at 3:1 ratio of medium to matrigel. 2 × 10^6^ cells per 100 *μ*L were then inoculated in the mouse prostate. Animals were sacrificed 9 weeks after inoculation. Primary tumors were isolated and weighted, and lymph node metastases were counted. Results were analyzed for statistical significance as previously described 26.

## Results

### Elevated Dyn2 expression levels are associated with early PCA development

**Figure 1:**
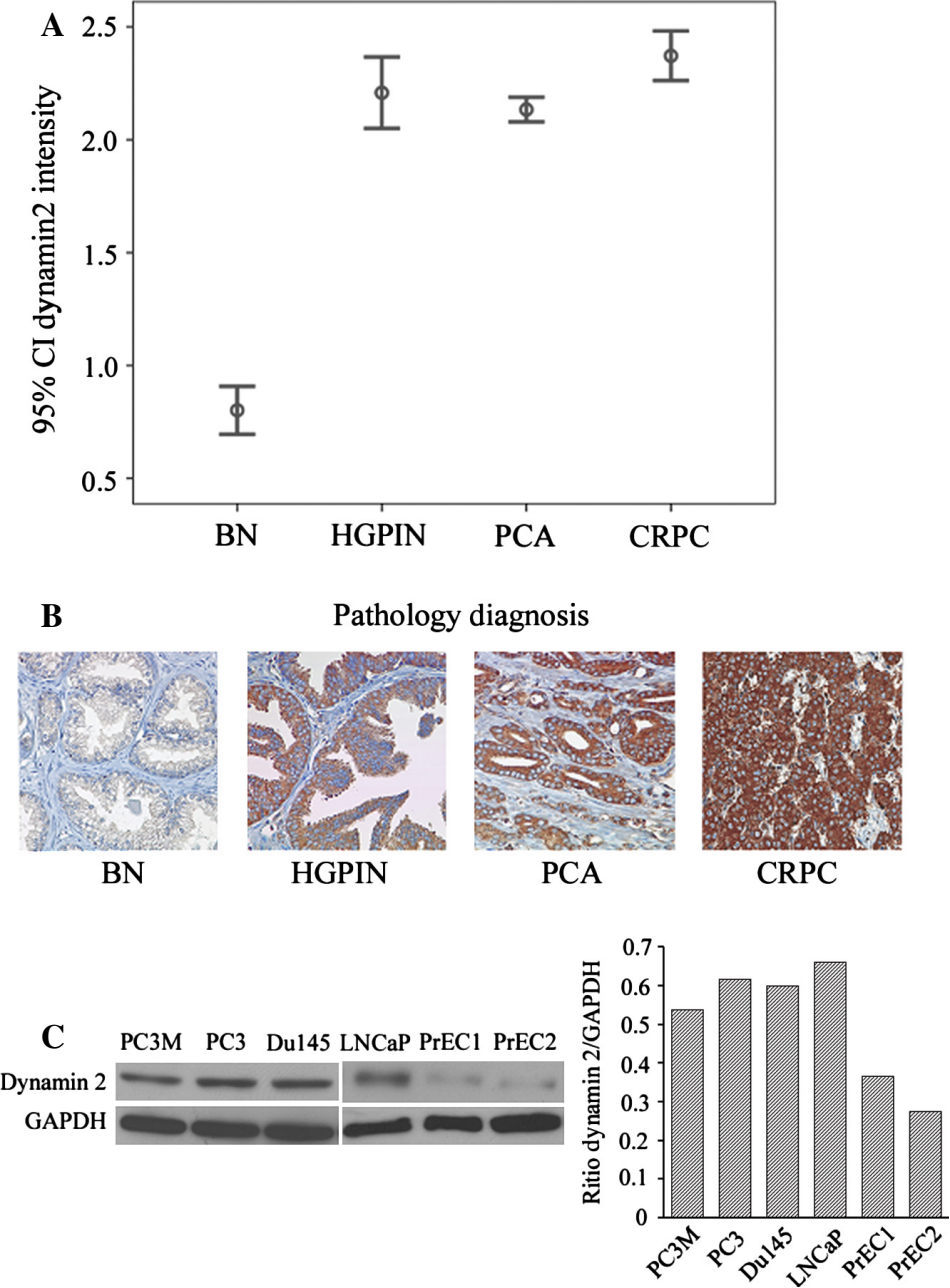
Dyn2 expression across different stages of PCA progression. (A) Dyn2 protein expression at different stages of PCA progression. Confidence intervals (95%) show normalized mean intensity value units of Dyn2 as determined by quantitative evaluation of immunohistochemistry. The *y*-axis represents numerical values corresponding to the intensity of Dyn2 expression (0, negative; 1, weak; 2, moderate and 3, high intensity). (B) Representative images of Dyn2 expression in adjacent benign prostate tissue (BN), HGPIN, PCA and CRPC. (C) Western blot analysis shows an overexpression of Dyn2 in PCA cancer cells (PC3M, PC3, DU145, LNCaP) compared to non-immortalized primary normal epithelial cells isolated from two independent origins (PrEC1 and PrEC2). The inserted graph represents the average densitometry values of Dyn2/GAPDH. PCA, prostate cancer; CRPC, castrate-resistant PCA; HGPIN, high-grade prostatic intraepithelial neoplasia. Dyn2, Dynamin 2; PrEC, prostate epithelial cells.

A total of 1780 cores were available for analysis. Samples included adjacent benign tissue (*n* = 192), HGPIN (*n* = 48), localized PCA (*n* = 942), and CRPC (*n* = 598). Immunohistochemistry staining showed a significant increase in Dyn2 expression in neoplastic epithelium compared to adjacent benign tissue. Mean intensity levels were lowest in BN (0.91 ± 0.78) and highest in CRPC (2.37 ± 0.78) (*P *<* *0.0001, Fig. 1A). Mean intensity levels in HGPIN and localized PCA were (2.21 ± 0.54) and (2.13 ± 0.78), respectively. Both expressions were significantly higher than those observed in adjacent BN (*P *<* *0.0001, Fig. 1B). However, differences between HGPIN and each of localized PCA or CRPC were not significant (*P *>* *0.5). Figure 1A–B illustrates intensity levels of Dyn2 expression across various stages of PCA progression. In agreement with immunohistochemistry data, upregulation of Dyn2 level was observed in isolated PCA cells compared to non-immortalized normal prostate epithelial cells (PrEC) (Fig. 1C).

### Elevated Dyn2 expression levels are associated with Gleason score, tumor volume, and PCA-specific mortality

**Figure 2:**
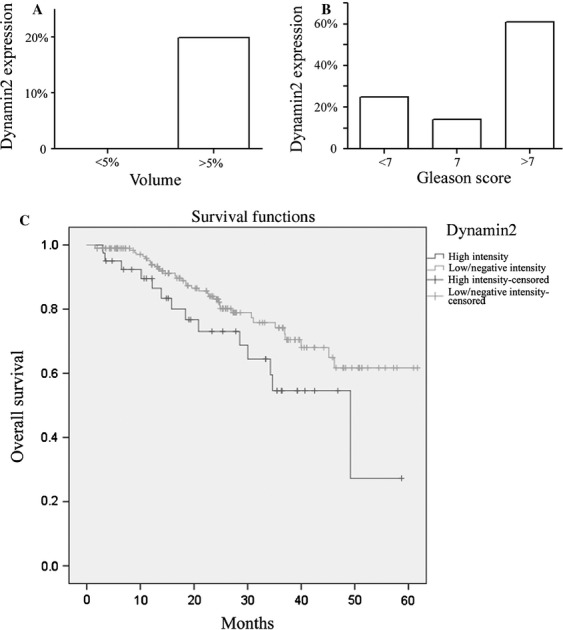
Differential expression of Dyn2 in relation to tumor size, Gleason score, and patients’ survival in the CRPC cohort. (A) Dyn2 protein expression levels distinguished patients with tumor stage volumes ≤5% vs. >5%, reflective of pT1a vs. pT1b in the TNM staging system for TURP samples (*P *=* *0.009). (B) Gleason score <7 vs. 7 and >7 (*P* = 0.004). (C) Kaplan–Meier curve demonstrating difference in the overall survival between high Dyn2 expression (moderate to high intensity) vs. low expression (negative to weak intensity) (log-rank test; *P* = 0.052). Dyn2, Dynamin 2; CRPC, castrate-resistant PCA.

Association between Dyn2 protein expression and Gleason score, tumor volume and cancer-specific mortality was analyzed using the CRPC cohort. As shown in Figure 2A, a significant association between high-Dyn2 protein expression and tumor volume of >5% (reflective of pT1a vs. pT1b stage within the TURP samples [*P *=* *0.009]) was observed, while a moderate to high Dyn2 expression was seen in the majority (∼90%) of PCA across different Gleason scores categories. High Dyn2 intensity was associated with higher Gleason score (*P *=* *0.004) (Fig. 2B). In this cohort of CRPC, 26.2% (38/145) patients with moderate to high Dyn2 expression had PCA cancer-specific mortality versus 15.6% (15/96) of patients with negative or weak Dyn2 expression (*P* = 0.052) (Fig. 2C). Using Cox regression model in this TURP cohort, incorporating Dynamin expression, GS and volume, only GS showed to be statistically significant (*P* = 0.003).

### Dyn2 regulates PCA cell invasiveness in vitro and in vivo

**Figure 3:**
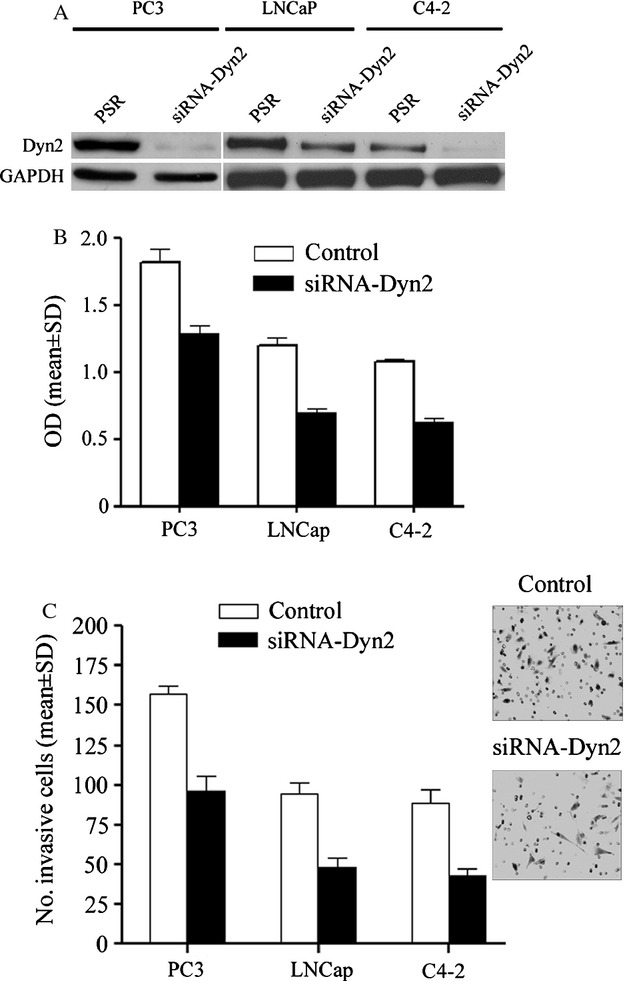
Impact of Dyn2 expression on PCA cell phenotypes. (A) Stable knockdown of Dyn2 in PCA cell lines. Representative Western blot image showing Dyn2 expression in the PCA cells stably engineered to express Dyn2 siRNA (siRNA-Dyn2) and their matched control cells (PSR). GAPDH was used as an internal control. (B) Downregulation of Dyn2 inhibits proliferation of both the androgen-responsive LNCaP cells and the androgen-non-responsive C4-2 and PC3 cells. Results are the average of four independent experiments. (C) Cell invasion was examined on PCA cells expressing PSR and Dyn2 siRNA using the Boyden chamber assay as described in Methods. The bar graph shows quantification of cell invasion results as the mean ± SD of four independent experiments and five fields per condition. Representative images of the most invasive PC3 cells are shown. PCA, prostate cancer; Dyn2, Dynamin 2.

To investigate the biological significance of Dyn2 to PCA progression, we generated clones of LNCaP, C4-2 and PC3 cells where Dyn2 was downregulated by stably expressing Dyn2 siRNA compared to control cells expressing scrambled siRNA (Fig. 3A). In the three cell models tested, inhibition of Dyn2 reduced cell proliferation (Fig. 3B) and cell invasion (Fig. 3C) in vitro. Similar results were also observed in independent cells expressing the Dyn2-dominant mutant, K44A (data not shown).

**Figure 4:**
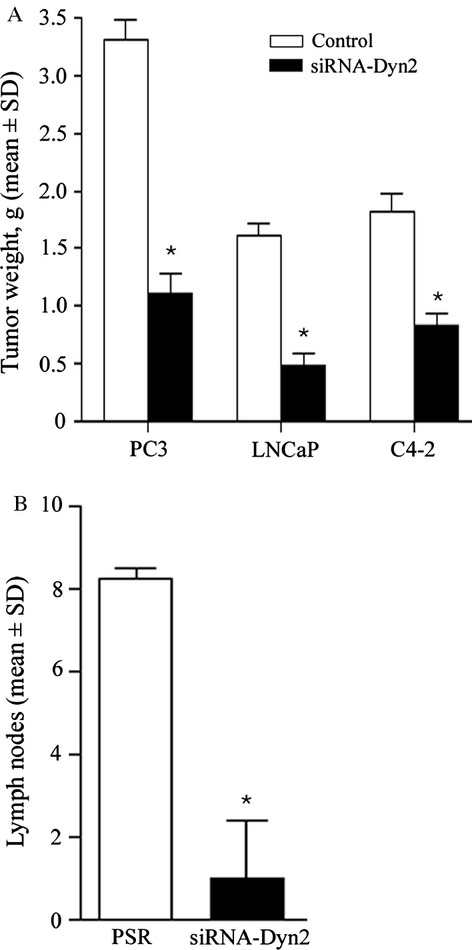
Inhibition of Dyn2 reduced tumor growth and cancer progression to metastases in androgen-responsive and androgen-refractory PCA mouse models. Cells expressing control scrambled siRNA (PSR) and Dyn2 siRNA (siRNA-Dyn2) were implanted into the prostate of SCID mice (*n* = 10). Animals were kept under observation for a period of 9 weeks to allow metastasis formation. Mice were then subjected to autopsy and primary tumors and lymph nodes were examined. Graphs show the average tumor size (A) or number of lymph nodes metastases (B) by mean ± SD. As noted, both tumor size (*P <* *0.01) and metastatic lymph nodes (*P <* *0.005) were reduced in Dyn2-silenced cells compared to PSR. PCA, prostate cancer; Dyn2, Dynamin 2.

In vivo, Dyn2 downregulation (siRNA-Dyn2) was achieved in the invasive and androgen receptor negative cell line PC3, and the androgen-responsive LNCaP and their matched androgen-resistant variant C4-2 cells. Implantation of these cells and their matched controls (PSR) into the prostates of mice (*n* = 10 per condition) revealed no difference in tumor uptake between control and Dyn2-knockdown groups. However, a significant decrease in tumor weights was noted in both androgen-resistant (3.313 ± 0.494 [PSR] vs. 1.112 ± 0.461 [siRNA-Dyn2] for PC3, and 1.82 ± 0.43 [PSR] vs. 0.832 ± 0.27 [siRNA-Dyn2] for C4-2) and the androgen-responsive LNCaP cells (1.618 ± 0.272 [PSR] vs. 0.492 ± 0.256 [siRNA-Dyn2]) (Fig. 4A). A significant reduction in lymph node metastases was noted in PC3 cells, the only cell line able to induce macroscopic lymph node metastases in our condition (Fig. 4B; the number of metastases in lymph nodes were 8.250 ± 0.240 [PSR] vs. 1.000 ± 1.410 [siRNA-Dyn2]).

### Downregulation of Dyn2 expression stabilizes FAs

**Figure 5:**
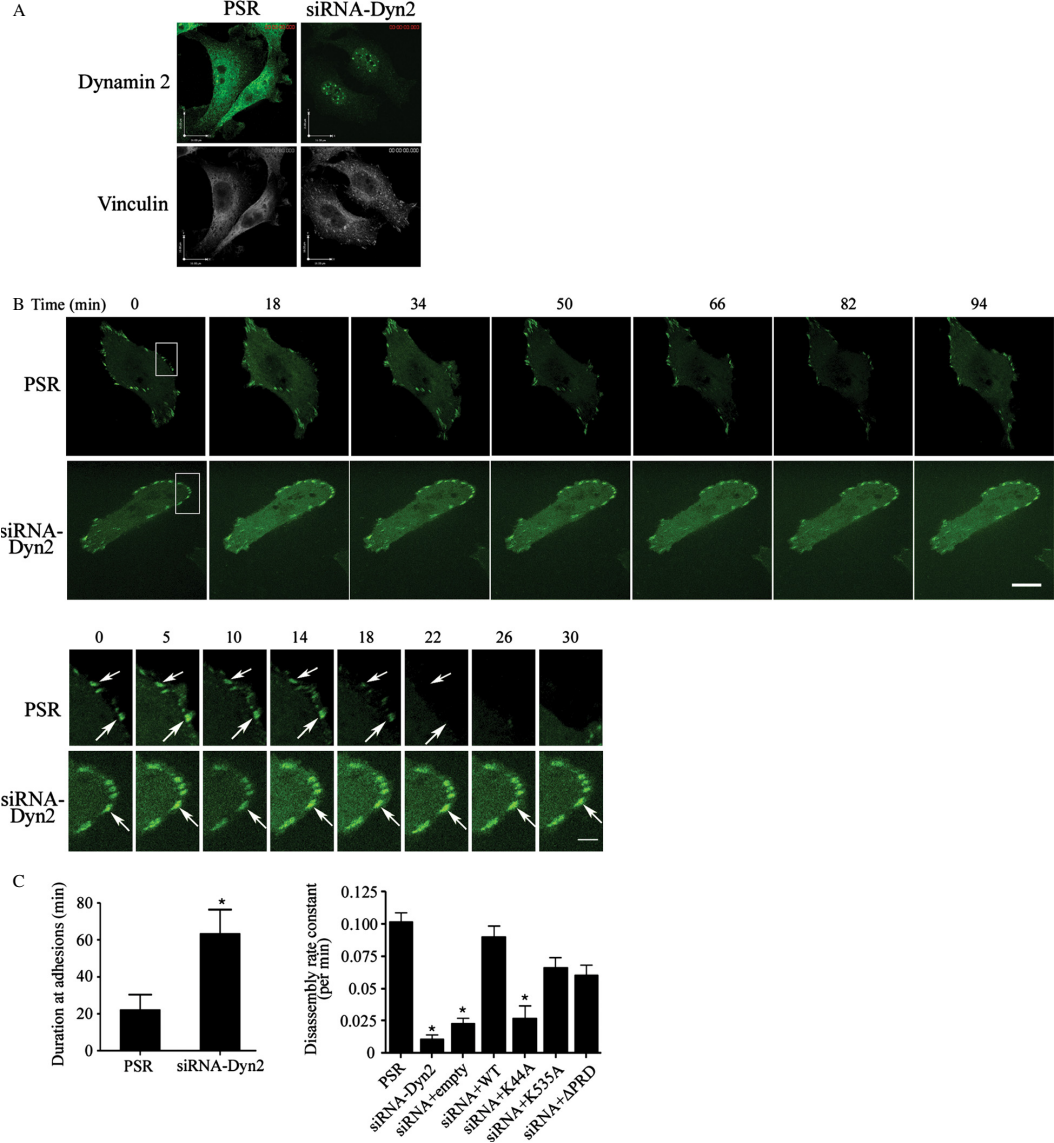
Inhibition of Dyn2 in PC3 cells stabilizes focal adhesions. (A) Representative immunofluorescence pictures showing FA structures using vinculin (FA marker) in control (PSR) and Dyn2-silenced PC3 cells (siRNA-Dyn2). Note that control cells exhibit a diffused expression of vinculin localized predominantly in the cytoplasm, whereas in Dyn2-silenced cells, FAs are numerous and prominently localized at the periphery of cell protrusions. Scale bar, 16 *μ*m. (B) Time-lapse quantification of FA dynamics after Dyn2 silencing. Control (PSR) and siRNA-Dyn2 cells transfected with EYFP-paxillin were analyzed using time-lapse spinning disk confocal microscopy as described in Methods. Representative frames are shown. Scale bar, 15 *μ*m. Enlargements of the boxed regions are shown below. The white arrows indicate positions of paxillin-containing FAs. Scale bar, 5 *μ*m. (C) Quantification of EYFP-paxillin persistence and turnover. EYFP-paxillin duration at FA in control and Dyn2 silenced (siRNA-Dyn2) PC3 cells were quantified as described in Methods. Dyn2 knockdown significantly prolongs the duration time at FA. Quantifications show the mean ± SD from three independent experiments (*P <* *0.01). FA turnover (expressed as FA disassembly rate constant per minute) was analyzed by time-lapse spinning disk confocal microscopy in siRNA-Dyn2 cells reconstituted with WT, K44A, ΔPRD, or K535A (*P <* *0.05). FA, focal adhesion; Dyn2, Dynamin 2; WT, wild type.

The impact of Dyn2 inhibition on PCA progression suggests an effect on cell invasion signaling. In particular, Dyn2 plays a major function in endocytosis of proteins involved in FA signaling, including integrin *β*-1 and FA kinase (FAK) 16,28. To examine this mechanism, fluorescence microscopy following immunostaining with anti-vinculin (a marker of FA), revealed a significant increase in the expression of vinculin at plasma membrane protrusions in siRNA-Dyn2 cells compared to matched control cells (Fig. 5A). Time-lapse confocal microscopy in live cells revealed that FA proteins remain stable for a prolonged period of time in Dyn2-silenced cells (siRNA-Dyn2) compared to control cells (Fig. 5B), in support of our previous study showing a correlation between high cell invasiveness and accelerated turnover of FA 29. Dyn2 protein is organized into a GTPase domain, middle domain, pleckstrin-homology (PH) domain, GTPase-effector domain, and PRD. To confirm the role of Dyn2 in FA turnover in PCA cells, FA disassembly rate was examined in Dyn2-silenced cells reconstituted with either rat WT Dyn2 or Dyn2 mutant deficient in GTPase, PRD, or PH domain. As shown in Figure 5C, reconstitution of Dyn2-silenced cells with Dyn2 mutants ΔPRD and K535A can partially restore FA turnover (*P *<* *0.05) and WT Dyn2 was necessary for full restoration of FA disassembly defect. These results support that the GTPase domain is necessary for Dyn2-induced FA turnover.

## Discussion

Dyn2, the most ubiquitous isoform of the dynamin family, exerts a broad physiological function in the regulation of vesicle transport and endocytosis trafficking 7. Herein, we demonstrate that Dyn2 expression is elevated in the majority of neoplastic prostate tissues compared to benign tissue and is associated with poor prognosis. The association of Dyn2 with early and advanced stages of PCA is similar to other PCA-associated biomarkers such as the ERG fusion gene 30. Our results are further supported by in vitro and in vivo studies in preclinical PCA models where Dyn2 inhibition resulted in a significant decrease in cell proliferation, and motility and invasion in both androgen-responsive and androgen-resistant PCA cell lines. As well a significant reduction in tumor progression was noted when PCA cells where Dyn2 was inhibited were implanted orthotopic into the mouse prostates compared to controls. Noticeably, a study using a small cohort of samples from pancreatic cancer reported a positive correlation between Dyn2 expression and malignancy although no comparison was made to clinical parameters such as outcome 31.

The molecular mechanisms by which Dyn2 contribute to cancer progression are partially understood. Of relevance to cell migration and invasion, Dyn2 has been shown to localize to FA, where it mediates the clathrin-dependent internalization of integrins 16,17. Moreover, a study by Wang et al. 28 demonstrated a role of Dyn2 in the regulation of FA turnover: they showed that Dyn2 is phosphorylated by Src kinase and then recruited to FA by a direct interaction with the FERM domain of FAK, which in turn regulates FA turnover as Dyn2 mutants disrupting this interaction fail to promote disassembly. In this context, we have reported the importance of FAK signaling for the regulation of cell invasion and FA turnover 23,27,29. In PCA cells, we observed that Dyn2 inhibition led to a drastic increase in FAs stability, which supports that Dyn2 silencing prevents FA rapid turnover, a perquisite for cell locomotion and migration. This study also demonstrates that the GTPase activity of Dyn2 is required for the regulation of FA turnover and cell motility in PCA cells. This has significance to future clinical studies with small molecules targeting Dyn2. Several Dyn2 inhibitors under development for clinical use have been found to inhibit cell proliferation and cancer cell migration 32,33.

While this study clearly points out to FA dynamics being an important target for Dyn2 signaling in invasive PCA cancer cells, we cannot rule out the contribution of other mechanisms independent from Dyn2-mediated FA turnover regulation. For instance, Dyn2 is critical for cell cycle progression and cell survival. As shown in our results, silencing Dyn2 in PCA cells reduced cell proliferation. However, analysis of apoptosis using the Annexin assay revealed no impact of Dyn2 silencing on cell apoptosis compared to control cells (data not shown). Moreover, Dyn2 is required for endocytosis of several oncogenic receptors such as EGFR and Her2 34,35, many of which have been implicated in prostate carcinogenesis. Dyn2 also regulates Golgi structure and vesiculation during the secretory process that can affect trafficking of other signaling molecules involved in carcinogenesis 8. Finally, Dyn2 directly interacts with F-actin and actin dynamics, actin-associated proteins, molecules that induce or sense membrane curvature, as well as expression of angiogenic receptors, e.g., vascular endothelial growth factor receptor 2/Kinase insert domain receptor, in endothelial cells 36,37, but future studies are required to delineate the impact of Dyn2 silencing on each of these mechanisms in progressive PCA. Taken together, this study provides a novel implication of Dyn2 signaling in PCA progression and support potential prognostic and therapeutic values of Dyn2 for advanced and hormone-refractory PCA.
